# Patterns of Hydroxyurea Prescription and Use in Routine Clinical Management of Polycythemia Vera: A Multicenter Chart Review Study

**DOI:** 10.4274/tjh.galenos.2020.2019.0431

**Published:** 2020-08-28

**Authors:** Yahya Büyükaşık, Rıdvan Ali, Mehmet Turgut, Güray Saydam, Akif Selim Yavuz, Ali Ünal, Muhlis Cem Ar, Orhan Ayyıldız, Fevzi Altuntaş, Müfide Okay, Rafiye Çiftçiler, Özgür Meletli, Nur Soyer, Metban Mastanzade, Zeynep Güven, Teoman Soysal, Abdullah Karakuş, Tuğçe Nur Yiğenoğlu, Barış Uçar, Ece Gökçen, Tülin Tuğlular

**Affiliations:** 1 Hacettepe University Faculty of Medicine, Department of Internal Medicine, Division of Hematology, Ankara, Turkey; 2 Uludağ University Faculty of Medicine, Department of Internal Medicine, Division of Hematology, Bursa, Turkey; 3 Ondokuz Mayıs University Faculty of Medicine, Department of Internal Medicine, Division of Hematology, Samsun, Turkey; 4 Ege University Faculty of Medicine, Department of Internal Medicine, Division of Hematology, İzmir, Turkey; 5 İstanbul University, İstanbul Faculty of Medicine, Department of Internal Medicine, Division of Hematology, İstanbul, Turkey; 6 Erciyes University Faculty of Medicine, Department of Internal Medicine, Division of Hematology, Kayseri, Turkey; 7 İstanbul University-Cerrahpaşa, Cerrahpaşa Faculty of Medicine, Department of Internal Medicine, Division of Hematology, İstanbul, Turkey; 8 Dicle University Faculty of Medicine, Department of Internal Medicine, Division of Hematology, Diyarbakır, Turkey; 9 Yıldırım Beyazıt University Faculty of Medicine, Department of Internal Medicine, Division of Hematology, Ankara, Turkey; 10 Dr. Abdurrahman Yurtaslan Ankara Oncology Training and Research Hospital, Division of Hematology, Ankara, Turkey; 11 Novartis Oncology, İstanbul, Turkey; 12 Marmara University Faculty of Medicine, Department of Internal Medicine, Division of Hematology, İstanbul, Turkey

**Keywords:** Polycythemia vera, Hydroxyurea, Treatment outcome

## Abstract

**Objective:**

This study aimed to evaluate real-life data on patterns of hydroxyurea prescription/use in polycythemia vera (PV).

**Materials and Methods:**

This retrospective chart review study included PV patients who had received hydroxyurea therapy for at least 2 months after PV diagnosis. Data were collected from 10 representative academic medical centers.

**Results:**

Of 657 patients, 50.9% were in the high-risk group (age ≥60 years and/or history of thromboembolic event). The median duration of hydroxyurea therapy was 43.40 months for all patients; 70.2% of the patients had ongoing hydroxyurea therapy at last follow-up. Hydroxyurea was discontinued in 22.4% of the patients; the most common reason was death (38.5%). The predicted time until hydroxyurea discontinuation was 187.8 months (standard error: ±21.7) for all patients. This duration was shorter in females (140.3±37.7 vs. 187.8±29.7) (p=0.08). This trend was also observed in surviving patients aged ≥50 years at hydroxyurea initiation (122.2±12.4 vs. 187.8±30.7, p=0.03). Among the patients who were still on hydroxyurea therapy, 40.3% had a hematocrit concentration of ≥45% at their last follow-up visit, and the rate of patients with at least one elevated blood cell count was 67.8%.

**Conclusion:**

Hydroxyurea prescription patterns and treatment aims are frequently not in accordance with the guideline recommendations. Its discontinuation rate is higher in females.

## Abstract (tr)

**Amaç:**

Polistemia vera’da (PV) hidroksiüre reçete etme/kullanma şekilleri hakkındaki gerçek yaşam verilerini değerlendirmek.

**Gereç ve Yöntemler:**

Bu retrospektif kayıt çalışmasına tanıdan sonra en az 2 ay hidroksiüre almış olan hastalar dahil edildi. Veriler ülkeyi temsil ettiği düşünülen 10 akademik tıp merkezinden toplandı.

**Bulgular:**

Altı yüz elli yedi hastadan %50,9’u yüksek risk (yaş ≥60 yıl ve/veya tromboembolik olay öyküsü) grubundaydı. Tüm hastalarda ortanca hidroksiüre tedavi süresi 43,4 aydı. Hastaların %70,2’si son kontrol tarihlerinde halen hidroksiüre kullanmaktaydı. Hidroksiüre en sık neden (%38,5) ölüm olmak üzere hastaların %22,4’ünde kesilmişti. Tüm hasta grubunda hidroksiüre kesmek için ortanca süre 187,8 ay ±21,7 standart hata olarak hesaplandı. Bu süre kadınlarda (140,3±37,7) erkeklere (187,8±29,7) göre daha kısa idi (p=0,08). Bu eğilim hidroksiüre başlandığında 50 yaş ve üstünde olan ve son durumda hayatta olan hastalarda da gözlendi (122,2±12,4 ve 187,8±30,7, p=0,03). Hidroksiüre devam etmekte olan hastalarda son kontrolde ≥%45 hematokrit %40,3 ve en az bir artmış kan hücre düzeyi %67,8 oranında gözlendi.

**Sonuç:**

Hidroksiüre reçete etme şekli ve tedavi hedefleri sıklıkla rehber önerilerine uymamaktadır. Hidroksiüre kesme oranı kadınlarda daha yüksektir.

## Introduction

Polycythemia vera (PV) is a Philadelphia chromosome-negative chronic myeloproliferative neoplasm. The incidence of PV has been estimated as 0.4-2.8 per 100,000/year in Europe and 0.8-1.3 in the United States [1]. The V617F mutation of the *Janus kinase* *2* (*JAK2*) gene on the 9^th^ chromosome is found in 95%-97% of PV patients and this discovery was a milestone in its diagnosis and treatment [2,3]. In many PV patients, the diagnosis can be overlooked for a long time and the disease may only be recognized after a thromboembolic event. Arterial and venous thromboses have been known as the major causes of morbidity and mortality in PV patients [2]. PV patients with advanced age (≥60 years) and/or with a history of a thrombotic event are defined as having high thromboembolic risk [4].

The primary goals of PV treatment are to relieve symptoms, to prevent thromboembolic complications without increasing the risk of bleeding, and to minimize the risks of leukemia and myelofibrosis as secondary events [2,5]. Current recommendations for initial treatment include phlebotomy and low-dose acetylsalicylic acid. Cytoreduction (generally hydroxyurea or interferon-alpha in standard or PEGylated forms) is also required in patients with high thromboembolic risk, in those intolerant to phlebotomy, and in those with disease-related symptoms refractory to palliative drugs and interventions. Second-line treatment (standard/PEGylated interferon-alpha or JAK inhibitor ruxolitinib) should be considered for patients with hydroxyurea resistance/intolerance [6,7].

Hydroxyurea has been used for the treatment of PV for nearly 40 years. Hydroxyurea therapy has been shown to decrease thrombotic event rates and leukemic transformation in PV patients [4,8]. It is the usual first-line treatment choice for cytoreduction based on long-term experience; moreover, it has the advantages of being an oral medication and having low treatment cost. On the other hand, there are also conflicting small studies reporting the increased risk of secondary solid cancer in patients with myeloproliferative neoplasms receiving hydroxyurea. Moreover, infertility and teratogenicity risks related to hydroxyurea should also be considered in young PV patients [8]. During recent years, the treatment aims of PV and the definitions of hydroxyurea resistance and intolerance have been clearly documented [9,10]. However, the impact of these developments on routine PV care has not been sufficiently documented. Thus, more data are required to show the patterns of hydroxyurea treatment of PV patients in daily practice and also the impact of these patterns on the treatment outcomes. This retrospective multicenter study aimed to evaluate real-life data on the patterns of hydroxyurea prescription and use in PV patients in Turkey.

## Materials and Methods

### Patients

This study included PV patients who were ≥18 years of age at the time of PV diagnosis and who either were currently on hydroxyurea therapy for at least 2 months or had received hydroxyurea therapy at least 2 months after PV diagnosis. Patients who were included in any other clinical trial for PV were excluded.

### Study Design

The present study, which was planned as a retrospective, descriptive, non-randomized study, involved the review of patient charts obtained from hematologists working in 10 study centers. The recruiting centers were selected from provinces with populations that were chosen as representative of the population of the relevant geographic region of Turkey. Although no formal sample size calculation was performed in the present study, the number of patients to be enrolled in each province was calculated based on the overall population and a maximum of 700 patients were expected to be enrolled. The patient charts were reviewed by the local investigators receiving assistance from a sponsored contract research organization (CRO). Statistical analyses were done by the principal author and a CRO statistician. The manuscript was written by the principal author and a CRO medical writer. All authors had rights to access the data and the final manuscript was sent to them. The study was sponsored by Novartis Pharmaceuticals Corporation. The study protocol was reviewed and approved by the Ethics Board of Hacettepe University.

### Procedure

The patient charts were reviewed retrospectively starting from the chart of the most recently examined patient and the ones fulfilling the selection criteria were included in the study. Data collection was performed through the transfer of the data from the patient charts to hard-copy data collection forms and then to electronic tables. Patients’ data regarding demographics, medical history, history of PV, presence of the *JAK2*V617F mutation, thrombosis and bleeding history, information on cytoreductive therapy, and findings of routine laboratory tests were collected from the charts.

### Definitions

Upfront treatment with hydroxyurea was arbitrarily defined as initiation of the drug within 2 months of diagnosis. High-risk disease was defined as age of ≥60 years and/or history of a thrombotic event [4].

Hydroxyurea resistance/intolerance was defined according to the European LeukemiaNet (ELN) criteria as follows: i) need for phlebotomy to keep the hematocrit concentration at <45% after hydroxyurea therapy used for at least 3 months at a dose of ≥2 g/day, or ii) uncontrolled myeloproliferation (platelet count of >400x10^9^/L and white blood cell [WBC] count of >10x10^9^/L) after hydroxyurea therapy used for at least 3 months at a dose of ≥2 g/day, or iii) failure to reduce massive splenomegaly (>10 cm underneath the rib) by at least 50% on palpation or failure to completely improve splenomegaly-related symptoms after hydroxyurea therapy used for at least 3 months at a dose of ≥2 g/day, or iv) an absolute neutrophil count of <1x10^9^/L or a platelet count of <100x10^9^/L or a hemoglobin level of <10 g/dL at the lowest hydroxyurea dose required to provide complete or partial clinicohematological response as defined by the ELN [9], or v) the presence of leg ulcers or other unacceptable hydroxyurea-associated nonhematological toxicities (mucocutaneous signs, gastrointestinal symptoms, pneumonia, or fever) [10].

### Statistical Analyses

Data analysis was performed using IBM SPSS Statistics for Windows, Version 23.0 (IBM Corp., Armonk, NY, USA). Descriptive statistics were expressed as numbers and percentages for categorical variables and as mean ± standard deviation or median (standard error [SE]) (range or interquartile range) for numerical variables. Normality of data was tested using visual (histograms, probability plots) and analytical methods (Kolmogorov-Smirnov/Shapiro-Wilk tests). The chi-square test was used to compare independent categorical variables, whereas Student’s t-test was used to compare numerical variables. The continuation rate of hydroxyurea was calculated as a ratio and also using Kaplan-Meier analysis. Values of p<0.05 were considered as statistically significant.

## Results

Charts of 657 PV patients who were diagnosed between January 1985 and July 2017 were reviewed until April 2018. Fifty-one patients not receiving hydroxyurea and 18 patients not fulfilling the criteria of the study protocol were excluded; accordingly, data of 588 patients were included in the statistical analyses. Of the patients, 56.8% were male, 44.9% were aged ≥60 years, 89.2% of the tested cases were positive for the *JAK2*V617F mutation, and 8.2% had an active or a history of a thromboembolic/vascular event at diagnosis. Of the patients, 50.9% (295/579) were in the high-risk group (age ≥60 years and/or history of a thrombotic event) at diagnosis. The general characteristics of the study patients and hydroxyurea therapy are presented in Tables 1 and 2, respectively.

All PV patients (n=588) received hydroxyurea therapy, while 1.7%, 2.4%, and 3.9% of the patients respectively received standard or PEGylated recombinant interferon alpha, or anagrelide as a second cytoreductive therapy in addition to hydroxyurea therapy (Table 2). It was observed that the most common initial hydroxyurea dosage was 500 mg BID (41%), followed by 500 mg QD (33.5%). Of the patients, 70.7% were observed to require at least one dose adjustment after initial dosing (Table 2).

Evaluation of the patients for whom the exact starting date of hydroxyurea treatment was known (n=496) revealed that there were 284 (57.3%) patients who were commenced on hydroxyurea therapy within the first 2 months after the diagnosis, whereas 212 (42.7%) patients started hydroxyurea later (at a median of 20.3 months [interquartile range: 7.1-49.5 months] after the diagnosis). The characteristics of the patients according to their hydroxyurea therapy schedule (i.e. upfront or later) are presented in Table 3.

High-risk patients were more frequently commenced on upfront therapy with hydroxyurea; the rates were 63% in the age group of ≥60 years (n=260) and 52.5% in the age group of <60 years (n=319) (p=0.019), and the rates were 72.3% in the patients with thrombotic/vascular event (n=54) and 55.6% in those without (n=523) (p=0.028). In addition, a WBC count of >15x10^9^/L was also associated with commencement of upfront hydroxyurea (the rates were 74% in those with a WBC count of >15x10^9^/L and 56.1% in those without; p=0.002).

The median duration of hydroxyurea therapy was 43.4 months (range: 2-378 months) for all patients. The number of patients who were still on hydroxyurea therapy at their last follow-up visit accounted for 70.2% (n=413) of the study population, whereas the number of those who discontinued hydroxyurea accounted for 22.4% (n=132). Excluding 43 cases with missing data, the discontinuation rate for hydroxyurea therapy was 24.2%. The median duration of hydroxyurea therapy was 36.4 months (range: 2-378 months) for the patients who discontinued hydroxyurea therapy and 45.8 months (range: 2-323 months) for those who continued.

The reported reasons for discontinuation of hydroxyurea therapy were death (38.5%), toxicity (33.3%), inappropriate disease control or disease progression (22.8%), and other/unknown (5.4%). The patients who discontinued and those who were continuing hydroxyurea treatment were compared according to their demographic/clinical variables. Gender was determined as a significant variable for discontinuation; the rate of discontinuation was significantly higher in females than in males (27.3% versus 16.3%, p=0.002) (Table 4). Limiting the analysis to patients aged ≥50 years, the rate of drug discontinuation was still higher among females (27.3% vs. 10.2%, p<0.001).

**Figure 1:**
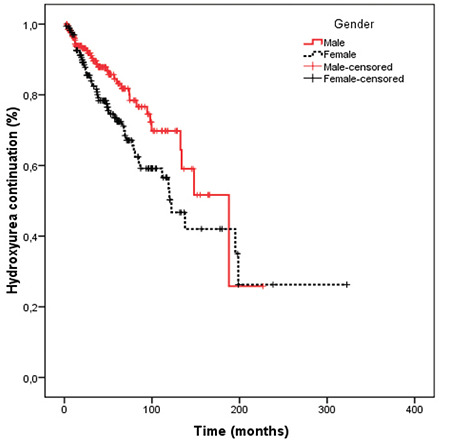
Kaplan-Meier analysis for continuation rate of hydroxyurea according to gender in surviving patients of ≥50 years of age.

According to the Kaplan-Meier analysis, the median time until hydroxyurea discontinuation was found as 187.8 months (SE: 21.7; range: 145.1-230.4 months) for all patients. This duration was shorter in females (140.3 months [SE: 37.7 months; range: 66.2-214.3 months]) than in males (187.8 months [SE: 29.7; range: 129.5-246 months]) (p=0.08). We also limited the Kaplan-Meier analysis to surviving patients aged ≥50 years at hydroxyurea initiation in order to understand if the main reasons for hydroxyurea discontinuation were fertility/teratogenicity concerns or death. We found that the median time until hydroxyurea discontinuation was still significantly shorter in females (122.2 months [SE: 12.4; range: 97.8-146.5 months] vs. 187.8 months [SE: 30.7; range: 127.5-248]) (p=0.03), indicating that the above-mentioned factors were not the main reasons for drug discontinuation (Figure 1).

Complete blood count values during the last follow-up visits of the patients still on hydroxyurea therapy are demonstrated in Table 5. Values indicating uncontrolled myeloproliferation were frequent, with rates of 40.3%, 29.2%, 34.4%, and 67.8% for elevated hematocrit (≥45%), platelet count (>400x10^9^/L), WBC count (>10x10^9^/L), and at least one of these three. The rates of hydroxyurea resistance (7.9%) and intolerance (8.1%) were estimated as 16% in total.

While the frequencies of thrombotic/vascular events, hemorrhage, and solid tumor at diagnosis were 8.2%, 2.2%, and 1.5%, respectively, they were 4.8%, 0%, and 0.17% under hydroxyurea therapy. Secondary myeloid neoplasms (i.e. acute myeloid leukemia or myelodysplastic syndrome) and post-PV myelofibrosis during hydroxyurea treatment were reported in 3 and 7 cases, respectively. The estimated risks at 10 years were 6.2% and 8%.

## Discussion

Hydroxyurea remains the first-line cytoreductive therapy in the treatment of patients with PV [11]. The present research was designed as a multicenter chart review study to evaluate the patterns of hydroxyurea prescription/use in PV patients in Turkey. Evaluation of real-life data is beneficial in order to obtain more information about patterns of therapy use, compliance with the guidelines, treatment outcomes, and reasons for failure and to establish strategies for improving therapeutic approaches.

PV is more prevalent in the elderly population and advanced age (≥60 years) is a significant risk factor for prognosis and survival. In our study, nearly half of the patients (44.9%) were ≥60 years. In a chart review study from Germany, 1476 PV patients were evaluated and 77.1% were found to be over the age of 60 years [12]. In a cross-sectional survey from Belgium, 343 PV patients were evaluated and 74.6% of them were ≥60 years of age [13]. Our cohort was relatively younger (median: 58 years), possibly due to the demographic characteristics of Turkey.

In their international study, Tefferi et al. [14] evaluated 1545 PV patients and reported advanced age and a leukocyte count of >15x10^9^/L as the risk factors for poor prognosis. The classical risk factors for thrombotic events in PV patients include age of ≥60 years, history of thrombosis, and a hematocrit concentration of ≥45%. In addition, other risk factors still in need of validation have been reported, such as an elevated WBC count, female gender, mutant *JAK2* allele burden, and classical cardiovascular risk factors [15]. In the present study, 50.9% of the patients were in the high-risk group (age ≥60 years and/or history of a thrombotic event).

Analysis of the *JAK2* mutation is an important criterion for the diagnosis of PV. In a prospective observational study from the United States, nearly half of the patients (n=2510) were investigated for the presence of *JAK2* mutation and 95.8% of these patients were found to be positive for *JAK2*V617F mutation [16]. In the present study, the *JAK2*V617F mutation was positive in 89.2% of the tested patients. The mutation status was unknown in 14.9% of the patients. These cases were mostly diagnosed before discovery of the mutation. Jentsch-Ullrich et al. [12] reported the mutation status as unknown in 23% of their patients, which was also attributed to the fact that the diagnoses were established before the molecular analysis became a routine part of the diagnosis. The *JAK2* mutation was included in the diagnostic criteria in the revised 2008 WHO classification system [17].

Although risk stratification of PV patients can be a guide for treatment, the patients may still be subjected to the risk of overtreatment or undertreatment [18]. A study evaluating real-life data demonstrated that cytoreductive drugs were not used in the majority of patients having an indication for cytoreductive therapy, also including high-risk patients [19]. Similarly, many of the high-risk patients in our cohort did not receive upfront therapy with hydroxyurea.

Patients with PV are at higher risk of morbidity and mortality as compared with the general population. Thrombotic and hemorrhagic events are the major complications associated with mortality [15]. Treatment focuses on prevention of these complications. It has been reported that patients treated with hydroxyurea had fewer vascular events as compared with those treated by phlebotomy alone [20]. Barbui et al. [21] compared hydroxyurea therapy with phlebotomy in PV patients and reported fewer cardiovascular events and hematological transformations (myelofibrosis transformation and acute leukemic transformation) as well as decreased overall mortality in those using hydroxyurea. In the present study, while the frequency of thrombotic/vascular event history at diagnosis was 8.2%, it was found to be 4.8% under hydroxyurea therapy.

Parasuraman et al. [22] reported that hydroxyurea was discontinued in 17.5% of their patients, mostly because of inadequate response (29.3%), intolerance (27.5%), and disease progression (12.7%). They also determined elevated blood cell counts (hematocrit concentration of ≥45%, platelet count of >400x10^9^/L, WBC count of >10x10^9^/L) in significant proportions of their patients on hydroxyurea (34.4%, 59.4%, and 58.2%, respectively). Similar outcomes were also obtained in the present study; hydroxyurea was discontinued in 22.4% of the patients and the main reported reasons for discontinuation were death (38.5%), toxicity (33.3%), and inappropriate disease control or progression (22.8%). Of the patients who were still on hydroxyurea therapy, 40.3% had a hematocrit concentration of ≥45%, 29.2% had a platelet count of >400×10^9^/L, and 34.4% had a WBC count of >10x10^9^/L.

Resistance/intolerance has been reported in nearly one-fourth of the patients receiving hydroxyurea therapy [15,23]. Hydroxyurea resistance/intolerance in PV patients is associated not only with an increased mortality rate but also with increased healthcare costs [24]. A study from Spain reviewed the medical charts of patients diagnosed with PV in 5 centers (n=261) and reported the frequencies of hydroxyurea resistance and intolerance as 11.5% and 12.6%, respectively [25]. In a larger series from Spain (n=890), the frequency of hydroxyurea resistance and/or intolerance was reported as 15% [26]. The difference between these two studies was attributed to different follow-up periods of patients or different doses of hydroxyurea used in the centers [26]. In a cross-sectional study from Belgium, 12% of the patients were reported to have hydroxyurea resistance/intolerance [13]. This rate was found to be 16% in the present study. However, we should accept that limited retrospective data quality (particularly the ambiguity about the definition of massive splenomegaly and the data concerning duration of hydroxyurea used at a dose of ≥2 g) might have obscured the exact figure. Among the patients who were still on hydroxyurea therapy in the present study, the rate of those with a hematocrit concentration of ≥45% at the last follow-up visit was 40.3%, and the rate of patients in whom at least one of the hematocrit, platelet, or WBC values was high was 67.8%. On the other hand, only 20.5% received ≥2 g/day hydroxyurea at any time during the disease course. This trend has not changed in recent years (data not shown). Accordingly, these data indicated that the ELN treatment response criteria [9] were not adequately considered and consequently hydroxyurea resistance was not routinely evaluated in clinical practice. Dose titration was not sufficiently considered despite elevated hematocrit concentration/blood count values under hydroxyurea therapy.

In the present study, discontinuation of hydroxyurea therapy was more common in females than in males (27.3% vs. 16.3%, p=0.002). Limiting the analysis to the patients aged ≥50 years, the drug discontinuation rate was still higher in females (27.3% vs. 10.2%, p<0.001). For this reason, we think that the higher hydroxyurea discontinuation rate in females was not due to fertility and/or teratogenicity concerns. We assume that aesthetic concerns due to cutaneous side effects might be a potential reason for this difference.

## Conclusion

The use of hydroxyurea for the treatment of PV in routine clinical practice is not in line with the guideline recommendations in some regards. Among hydroxyurea users, a substantial proportion of patients had blood count values that remained higher than recommended in the guidelines. These findings underline the need for more emphasis on the importance of reviewing treatment algorithms in accordance with the recommendations of current guidelines and following these algorithms in clinical practice. The finding of a higher hydroxyurea discontinuation rate among women and its reasons should be further investigated.

## Figures and Tables

**Table 1:**
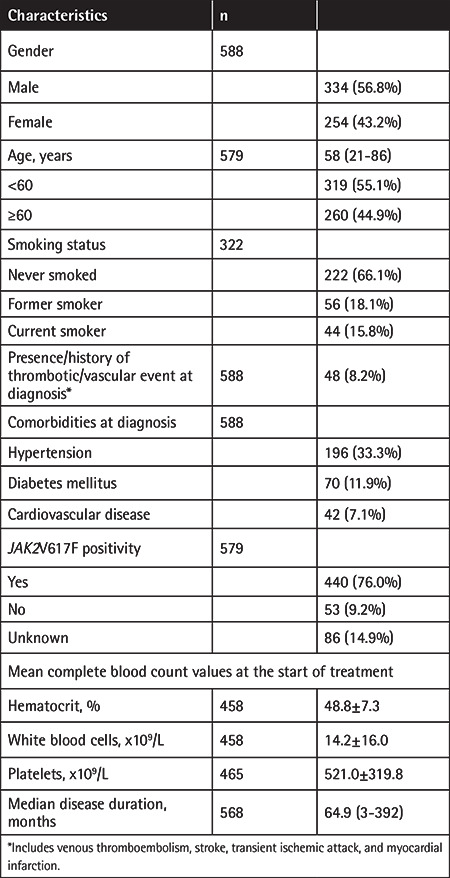
General characteristics of the patients.

**Table 2:**
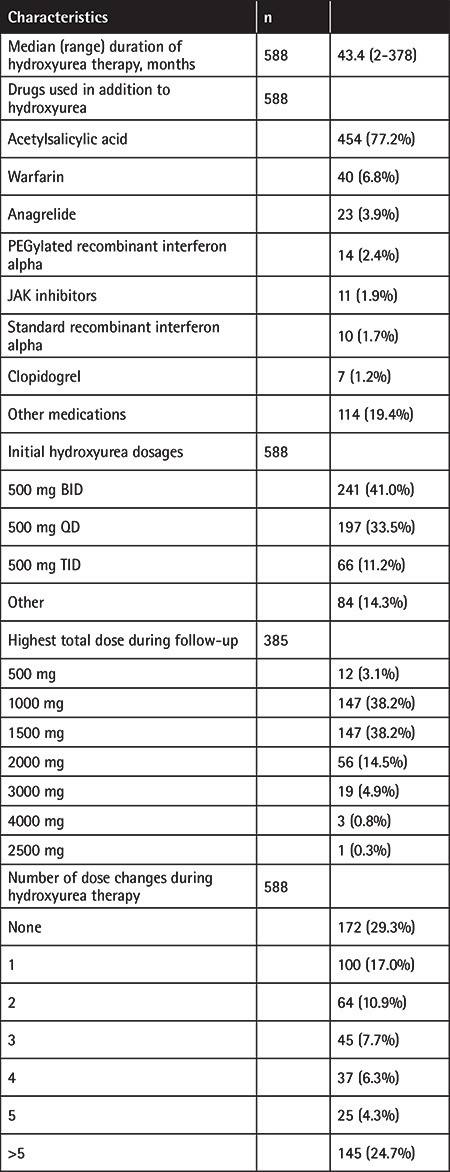
General characteristics of hydroxyurea therapy.

**Table 3:**
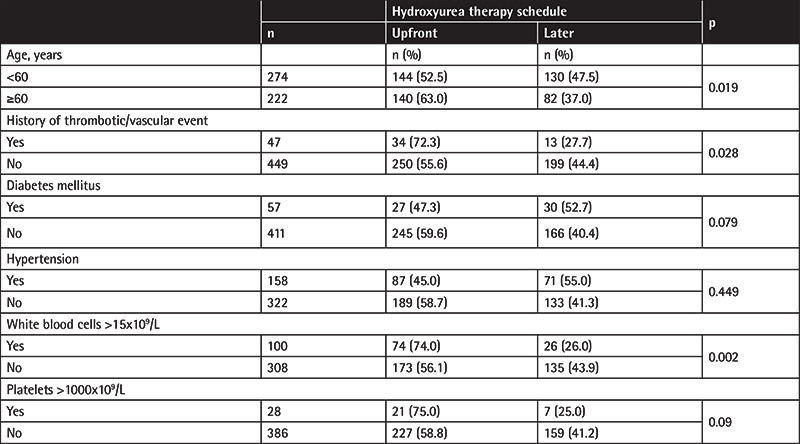
Characteristics of the patients according to their hydroxyurea therapy schedule.

**Table 4:**
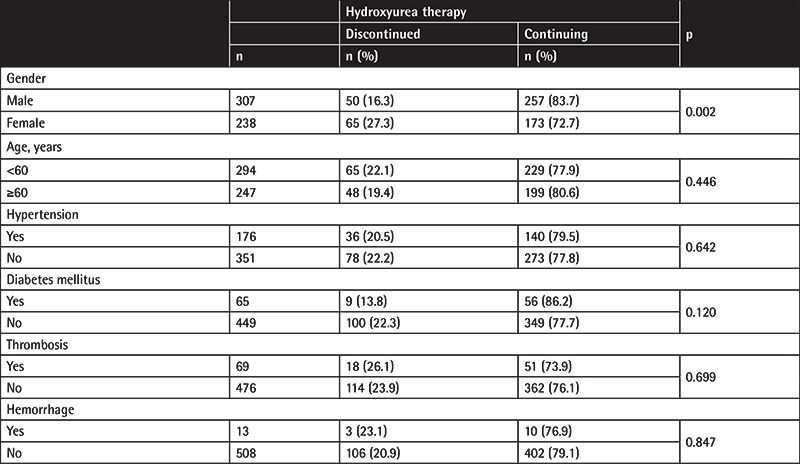
Comparison of the patients who discontinued or were continuing hydroxyurea therapy according to demographic/clinical variables.

**Table 5:**
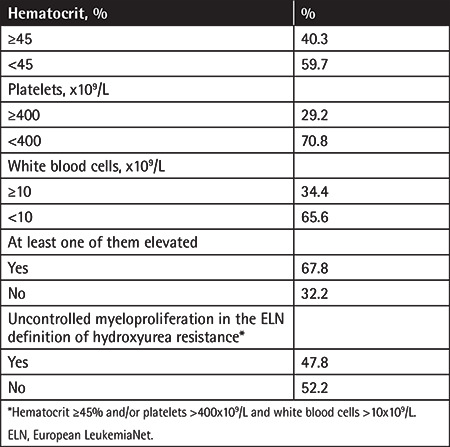
Complete blood count values during the last followup visits of the patients who were still on hydroxyurea therapy.
